# Review of interventions for apathy among persons living with dementia: international comparison

**DOI:** 10.1093/geroni/igaf122.4239

**Published:** 2025-12-31

**Authors:** Mariya Kovaleva, Peace Johnson, Cindy Schmidt

**Affiliations:** University of Nebraska Medical center, University of Nebraska Medical Center College of Nursing, Nebraska, United States; University of Nebraska Medical Center, University of Nebraska Medical Center College of Nursing, Nebraska, United States; University of Nebraska Medical Center, Leon S. McCoogan Health Sciences Library, Nebraska, United States

## Abstract

Apathy is the most prevalent neuropsychiatric symptom among persons living with dementia (PLWD). Caregivers frequently struggle with providing appropriate and engaging activities for their PLWD. Lack of appropriate activities also characterizes hospitals and rehabilitation facilities, undermining PLWDs’ quality of life, dignity, and personhood. Apathy differs from depression. Despite its prevalence, little is known about efficacious interventions for apathy. We aimed to conduct a scoping review of the literature on evidence-based interventions for apathy developed in the U.S. and internationally in 2000-2025 and to compare these interventions. Specifically, we aimed to describe the intervention details, populations for whom they are offered (e.g., diagnosis, residence types); and outcomes for PLWD, caregivers, and healthcare systems. Guideline-directed (Arksey & O’Malley, 2005) scoping review of the literature. Librarian conducted the literature search using six databases: Medline, PsycINFO, CINAHL, Scopus, EMBASE, and Cochrane Library. Six hundred and thirty-four U.S. articles and 1375 non-U.S. articles were retrieved. Peer-reviewed articles describing intervention testing are selected. Full results with international comparison are currently pending. Preliminarily, we identified 18 relevant articles on the U.S. interventions. Results encompassing interventions developed internationally will be ready by the time of the presentation, if selected. Although dementia is incurable, neuropsychiatric symptoms are modifiable. Non-pharmacological approaches are the mainstay in addressing neuropsychiatric symptoms due to little to no adverse effects, as opposed to pharmacologic management of symptoms. Healthcare organizations and community agencies must prioritize interventions for apathy to uphold PLWDs’ dignity and potentially decrease caregiver burden. Such interventions may be feasible for many organizations.

